# Surgical Reports and Miscellaneous Papers on Medical Subject

**Published:** 1855-08

**Authors:** 


Art. V.—*Surgical Reports and Miscellaneous Papers on Medical Subjects.* By George Hayward, M. D., President of the Massachusetts Medical Society; late Professor of Surgery in Harvard University, etc., etc. Boston: Phillips, Sampson & Company. New York: J. C. Derby, 1855; 8vo.; pp. 452.

Dr. Hayward stands among the foremost of the medical profession in Boston—a city celebrated for its skillful physicians and learned surgeons. His professional life covers a period of forty years. Throughout the whole of that time he has been a conscientious follower of his calling—throughout the greater part of it he has been a laborious and manful worker. In the evening of his well-spent days, when, as he expresses it, he "did not feel capable of much mental effort," he has occupied himself in reviewing his professional life, and the result of it has been the presentation of the volume before us. In thus collecting in one handsome sheaf the products of his pen, which before were scattered over the pages of our periodic literature, Dr. Hayward has conferred a lasting favor upon the profession. He has furnished a work which may be read with advantage and consulted with profit by all. It abounds in facts and tables; is replete with sound, practical precepts, and rich in prudent counsel.

For some years past the medical publishing of the country has been so exclusively in the hands of New York and Philadelphia houses, that we had almost ceased to look on the title-page for the names of any other firms than those who flourish in those cities. We hope in future that either the profession in Boston will write more, or that authors elsewhere will more frequently bring their works out under the auspices of Phillips, Sampson & Company; for no more pleasant book to the hand or eye was ever issued than the present volume. It is for sale in this city at the bookstore of Maxwell & Co. D. W. Y.

